# An investigation into anti-proliferative effects of microRNAs encoded by the miR-106a-363 cluster on human carcinoma cells and keratinocytes using microarray profiling of miRNA transcriptomes

**DOI:** 10.3389/fgene.2014.00246

**Published:** 2014-08-25

**Authors:** Cuong Khuu, Anne-Marthe Jevnaker, Magne Bryne, Harald Osmundsen

**Affiliations:** ^1^Department of Oral Biology, University of OsloOslo, Norway; ^2^Norwegian Scientific Committee for Food Safety (Government, Governmental)Oslo, Norway

**Keywords:** miR-17-92, miR-106-363, miR-106b-25, miR-20b, miR-92a, miR-363-5p proliferation, cancer

## Abstract

Transfection of human oral squamous carcinoma cells (clone E10) with mimics for unexpressed miR-20b or miR-363-5p, encoded by the miR-106a-363 cluster (miR-20b, miR-106a, miR-363-3p, or miR-363-5p), caused 40–50% decrease in proliferation. Transfection with mimics for miR-18a or miR-92a, encoded by the miR-17-92 cluster (all members being expressed in E10 cells), had no effect on proliferation. In contrast, mimic for the sibling miRNA-19a yielded about 20% inhibition of proliferation. To investigate miRNA involvement profiling of miRNA transcriptomes were carried out using deoxyoligonucleotide microarrays. In transfectants for miR-19a, or miR-20b or miR-363-5p most differentially expressed miRNAs exhibited decreased expression, including some miRNAs encoded in paralogous miR-17-92—or miR-106b-25 cluster. Only in cells transfected with miR-19a mimic significantly increased expression of miR-20b observed—about 50-fold as judged by qRT-PCR. Further studies using qRT-PCR showed that transfection of E10 cells with mimic for miRNAs encoded by miR-17-92 - or miR-106a-363 - or the miR-106b-25 cluster confirmed selective effect on expression on sibling miRNAs. We conclude that high levels of miRNAs encoded by the miR-106a-363 cluster may contribute to inhibition of proliferation by decreasing expression of several sibling miRNAs encoded by miR-17-92 or by the miR-106b-25 cluster. The inhibition of proliferation observed in miR-19a-mimic transfectants is likely caused by the miR-19a-dependent increase in the levels of miR-20b and miR-106a. Bioinformatic analysis of differentially expressed miRNAs from miR-106a, miR-20b and miR-363-5p transfectants, but not miR-92a transfectants, yielded significant associations to “Cellular Growth and Proliferation” and “Cell Cycle.” Western blotting results showed that levels of affected proteins to differ between transfectants, suggesting that different anti-proliferative mechanisms may operate in these transfectants.

## Introduction

**Figure 1:**
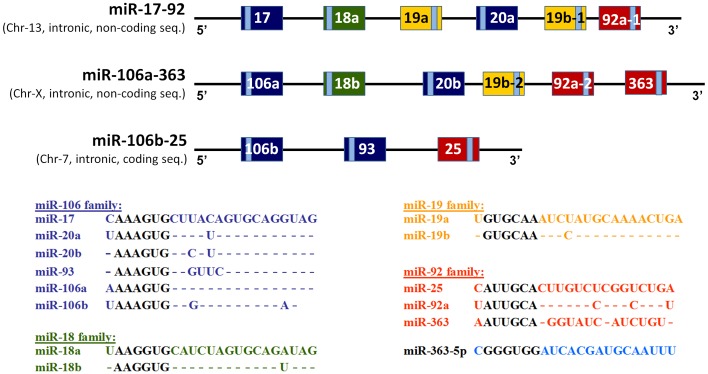
**Schematic illustration of structures of the three paralogous miR-17-92-, miR-106a-363-, and miR-106b-25 clusters**. The structure of the genes encoding three paralogous, polycistronic, miRNA clusters: the miR-17-92 cluster, the miR-106a-363 cluster and the miR-106b-25 cluster. The resulting mature miRNAs have been partitioned into four groups according to degrees of homologies of their seed sequences. The light-blue colored rectangles indicate positions of the mature miRNAs within the pre-miRNA structures.

MicroRNAs (miRNAs) are associated with regulation of biological phenomena ranging from cancer (for review see Lee and Dutta, 2009) to cardiac ischemic tolerance (Dharap and Vemuganti, 2010). Some miRNAs are transcribed from a single polycistronic transcript, e.g., the miR-17-92 cluster and its two paralogs, the miR-106a-363 and miR-106b-25 clusters (Bartel, 2004; He et al., 2005; Zhang et al., 2009). Fifteen miRNAs encoded by these three clusters can be separated into four families based on their seed sequences (Figure 1). The miR-17-92 cluster, located on human chromosome 13, encodes six miRNAs: miR-17, miR-18a, miR-19a, miR-20a, miR-19b-1, and miR-92-1. The miR-106a-363 cluster, located on human chromosome X, encodes six miRNAs: miR-106a, miR-18b, miR-20b, miR-19b-2, miR-92-2, and miR-363. The miR-106b-25 cluster, located on human chromosome 7, encodes three miRNAs: miR-106b, miR-93, and miR-25. While the miR-17-92 and miR-106a-363 clusters reside in introns of non-coding genes, the gene encoding miR-106b-25 is located in intron 13 of *MCM7* (Mendell, 2008).

High levels of expression of members of the miR-17-92 cluster have been observed in tooth germs, in developing tissues and in carcinoma cells (Lu et al., 2007; Jevnaker and Osmundsen, 2008; Jevnaker et al., 2011). Knock-out of this cluster caused defective embryonic development and early post-natal death (Ventura et al., 2008), while mice with knock-out for the miR-106b-25 cluster and/or the miR-106a-363 cluster exhibited no marked changes in phenotype (Ventura et al., 2008). In mice the miR-106a-363 cluster is normally not expressed except following retroviral integration at the Kis2 locus (Landais et al., 2007).

Substantial inter-dependence between levels of microRNAs and activity of signaling pathways is likely (Avraham and Yarden, 2012), as exemplified by miRNA-dependent regulation of cell cycle and of apoptosis (Jovanovic and Hengartner, 2006; He et al., 2011). Our preliminary studies had revealed that, among members of the miR-106a-363 cluster, only miR-106a was detectable in human cultured squamous carcinoma cells (clone E10), while all sibling miRNAs from the paralogous cluster were expressed. This observation elicited a study of effects of transfection with mimics for unexpressed members of the miR-106a-363 cluster on E10 cells. Effects on proliferation and apoptosis were studied using flow-cytometry, while effects on miRNA transcriptomes were investigated using microarrays and qRT-PCR. The results suggested that these miRNAs significantly diminished proliferation of E10 cells. Also, in all transfectants investigated distinct populations of differentially expressed miRNAs were found, although bioinformatic analysis revealed these populations to exhibit significant associations with identical cellular functions (e.g., “Cell Cycle,” “Cell Death” and “Cellular Growth and Proliferation”). The more highly expressed siblings, miR-19a and 92a, were used as “controls.”

Follow-up studies using qRT-PCR revealed that the “anti-proliferative” miR-363-5p markedly decreased expression of all other miRNAs in the three clusters. Although transfectants for both miR-19a -and miR-20b-mimic exhibited diminished proliferation expression of only a few sibling miRNAs were now significantly changed, but in a manner conducive to diminished proliferation.

## Materials and methods

### Cell lines

Human squamous carcinoma cell lines [clone E10 (PE/CA-PJ49) and clone C12 (PE/CA-PJ34)], were obtained from the European Collection of Cell Cultures (ECACC). Human colon cancer cell lines (HTC116 and HT29) were purchased from the American Type Culture Collection (ATCC). The cells were grown in Iscove's Modified Dulbecco's Medium (Sigma-Aldrich, St. Louis, MO, USA), supplemented with 10 % (v/v) FBS, 2 mM L-glutamine, 1 % (v/v) penicillin, 1% (v/v) streptomycin and 0.25 μg/ml amphotericin B (all reagents purchased from Lonza, Basel, Switzerland).

Primary human normal oral keratinocytes (KCs) were isolated from biopsy samples after third molar extraction (13). The procedure had been approved by the Ethics Committee in Norway. Primary KC and HaCaT cells (a gift from Dr. Eshrat Babaie, The Biotechnology Centre, University of Oslo) were grown in KC serum free medium (KSFM) supplemented with 1 ng/ml human recombinant epidermal growth factor and 25 mg/ml bovine pituitary extract (GibcoBRL, Dorset, UK). The medium was further added 1% (v/v) penicillin, 1% (v/v) streptomycin, and 0.25 mg/ml amphotericin B (Lonzo, Basel, Switzerland). The cells were split twice weekly at 80% confluence. Only freshly isolated KC cells were used experimentally.

### Transfection of the E10 cells with miRNA mimics or with scrambled control

Cells (E10) were incubated in IMDM medium with 5% FBS and transfected using INTERFERin (Polyplus-Transfection, Illkirch, France) according to the manufacturer's protocol. Transfections were carried out using 20 nM of miR-19a -, miR-20b -, or miR-92a -, or miR-363-3-p -, or miR-363-5-p mimic or Allstar scrambled control (Qiagen, Hilden, Germany), or with transfection reagent only (MOCK). All miRNA mimics were purchased from GenePharma, Shanghai, China. Transfection efficiencies were evaluated by flow-cytometry using the FACSCalibur flow-cytometer (Becton Dickinson, Heidelberg, Germany) with FITC-labeled scrambled RNA, and found >95% effective.

### Imaging and counting of cells in culture

At 72 h post-transfection the cell cultures were photographed using a Zeiss Axiovert 25 microscope at 5x magnification (Carl Zeiss, Jena, Germany). Following trypsination, cells were counted using Beckman Coulter Z2 (Beckman Coulter, Inc., Fullerton, CA, USA).

### Assay for apoptosis or proliferation using flow cytometry

Transfected cells were assayed for apoptosis and cell death by flow-cytometry using the Single Channel Annexin V/Dead Cell Apoptosis Kit with Alexa Fluor® 488 annexin V and SYTOX® Green for Flow Cytometry (Invitrogen, Carlsbad, CA, USA). At 72 h post-transfection cells were harvested by trypsination according to the manufacturer's protocol. About 500.000 cells were incubated in 200 μl annexin-binding buffer in the presence of Alexa Fluor 488 annexin V (Component A) and 50 nM SYTOX Green for 30 min prior to analysis. Floating cells, which constituted <10% of the total number of cells, were also included in the assay. E10 cells incubated with mitomycin C (10 μg/ml) for 20 h prior to analysis were used as positive control.

Cell proliferation was assayed using the Click-iT EdU Alexa Fluor® 488 assay kit (Life Technologies Ltd, Paisley, UK) as recommended by the manufacturer. E10 cells were incubated with 15 μM EdU for 2 h prior to fixation and labeling for analysis.

The assays were carried out using the FACSCalibur flow-cytometer equipped with CellQuest software.

### Selected mRNA targets and proteins for QRT-PCR and western blotting

Using MicroCosm (http://www.ebi.ac.uk/enright-srv/microcosm/cgi-bin/targets/v5/search.pl) KRT14 was found to be targeted by miR-363-5p. Target search using Ingenuity Pathway Analysis (IPA, http://www.ingenuity.com) suggested KRT15 to be targeted by miR-363-5p. SVMicro (Liu et al., 2010) suggested ATF1, KRT14, KRT15, PSMB6 as potential targets for miR-20b and miR-363-5p. IPA and SVMicro also suggested EGFR as target for miR-20b.

Preliminary mRNA profiling using microarrays (results not shown) had suggested that ATF1, KRT14, KRT15, PSMB6 were differentially expressed in miR-363-5p transfectants and AFT1 and PSMB6 in miR-20b transfectants. Levels of ATF1, KRT14, KRT15, PSMB6, and EGFR were also assayed using qRT-PCR.

Levels of expression were normalized using the mRNA encoding the TATA-binding protein (TBP) as house-keeping gene as this gene was found to exhibit constant levels of expression. The required primers were designed using Primer 3 software (Rozen and Skaletsky, 2000). The primers used were: ATF1; ATF1-f: TCTGGAGTTTCTGCTGCTGT, ATF1-r: ACTGTAAGGCTCCATTTGGG), EGFR; EGFR-f: GGAGAACTGCCAGAAACTGACC, EGFR-r: GCCTGCAGCACACTGGTTG, KRT14; KRT14-f: GGCCTGCTGAGATCAAAGAC, KRT14-r: TCTGCAGAAGGACATTGGC, KRT15; KRT15-f: AGCCCAGAATGCGACTACAG, KRT15-r: GCATTGTCGATCTCCAGGAT, PSMB6; PSMB-f: AACCACTGGGTCCTACATCG, PSMB6-r: CAGCTACTGCCTGGGTATCA, TBP; TBP-f: CGTGGCTCTCTTATCCTCATGA, TBP-r: GCCCGAAACGCCGAATATA.

The High-Capacity cDNA Reverse Transcription Kit was used to reversely transcribe mRNAs contained in 2 μg of DNase-treated total RNA using random primers (Applied Biosystems, CA, USA). Each reverse transcription reaction (total volume of 20 μl) was prepared according to the manufacturer's protocol and run in an Eppendorf MasterCycler (Eppendorf GmBh, Hamburg, Germany). The Stratagene MX 3005 qRT-PCR instrument was used together with the SYBR Green green-based qRT-PCR core kit (Eurogentec, Seraing, Belgium). Every sample was assayed in technical and biological triplicates.

### Isolation of RNA

Total RNA was extracted 72 h after transfection from E10 cells transfected with miRNA mimics or scrambled control, and from C12, HTC116, and HT29 cells, using the RNeasy Mini Kit as described by the manufacturer (Qiagen, Hilden, Germany). The RNA samples were treated with the TURBO DNA-free kit (Life Technologies Ltd, Paisley, UK) to remove contaminating genomic DNA.

RNA-fractions enriched with respect to miRNAs were isolated according to the manufacturer's protocol from transfected E10 cells using the mirPremier microRNA isolation kit (Sigma-Aldrich, St. Louis, MO, USA).

Concentrations of solutions of purified RNA were assayed using the Nanodrop ND-1000 spectrophotometer. RNA fractions exhibiting a ratio of OD260/OD280 and OD260/OD230 of at least 1.8 and 2.0, respectively, were used for further analysis.

### Microarray mapping of miRNAs exhibiting significantly changes in level of expression

MiRNAs with significantly altered expression following transfection with mimics for miR-19a, miR-20b or miR-363-5p on miRNA were identified using human deoxyoligonucleotide microarrays for miRNA (OneArray® Microarray v2). The miRNA arrays contain 100% of Sanger miRBase v15 miRNA content, i.e., 1087 unique miRNA probes. All miRNA probes were printed in triplicates. MiRNA-enriched fractions (about 500 ng of RNA), isolated with mirPremier microRNA kit, were labeled with Cy3 or Cy5 using Kreatech ULS labeling of miRNA (Kreatech, Amsterdam, Netherlands).

Hybridization of labeled miRNA was carried out at 41°C for 18 h in a SlideBooster 400 Hybridization Station (Advalytix, Munich, Germany). Microarray analysis was carried out in technical and biological triplicates. The microarrays were scanned in a Packard Bioscience Scanarray Lite microarray scanner (Perkin-Elmer, Waltham, MA, USA). The Cy3 and Cy5 fluorescence signals were quantified by using the ScanArrayExpress v.3.0 software (Perkin-Elmer, Waltham, MA, USA). All microarray data have been have been deposited in the ArrayExpress database with reference E-MTAB-1395.

### Statistical analysis of microarray data

Fluorescence-intensities were filtered using following criteria: only miRNAs with consistent fluorescence-intensities for each set of triplicate spots were used, and spots with net median fluorescence intensities of less than twice median background fluorescence intensity were excluded from further analysis. The statistical analysis was carried out using SPOTFIRE v. 9.0 DecisionSite for Microarray Analysis software (TIBCO Software, Palo Alto, CA, USA).

Microarray data were subjected to single channel analysis using log_2_ net fluorescence-intensities (median values, with background subtracted) normalized by scaling from 0 to 1. False discovery rates (FDR) computed according to Hochberg and Benjamini (1990) were used to correct selection of genes for false positives (*P* < 0.05). Normalization between arrays was carried out using Z-score normalization. Statistical analysis of microarray data was carried out on data derived from triplicate slides derived from three separate transfections. Results from batches of slides were combined into a single data-file for statistical analysis and subjected to ANOVA using SPOTFIRE software to isolate differentially expressed miRNAs (*p* ≤ 0.05).

### Reverse transcription of miRNAs

TaqMan® MicroRNA Assay (hsa_miR-106a, hsa_miR-106b, hsa_miR_17^*^, hsa_miR_17, hsa_miR_18a, hsa_miR-18b, hsa_miR_19a, hsa_miR-19b, hsa_miR_20a, hsa_miR-20b, hsa_miR-25, hsa_miR_92a, hsa_miR-93, hsa_miR-363, miR-363-5p, and miR-485-5p) and TaqMan® Control Assay (RNU48) were used to reversely transcribe 40 ng of RNA, enriched with respect to miRNA, to cDNA (AppliedBiosystems, Carlsbad, CA, USA). Each 15 μl reverse transcription reaction was performed according to the manufacturer's protocol and run in an Eppendorf MasterCycler (Eppendorf GmbH, Hamburg, Germany).

### Quantitative PCR (qRT-PCR)

TaqMan® MicroRNA qRT-PCR assay was used to validate microarray results and to further investigate effects of transfection with miRNAs-mimics on levels of paralogous miRNAs. Using the TaqMan® Universal PCR Master Mix (AppliedBiosystems, Carlsbad, CA, USA) the following assays were carried out: hsa_miR-106a, hsa_miR-106b, hsa_miR_17^*^, hsa_miR_17, hsa_miR_18a, hsa_miR-18b, hsa_miR_19a, hsa_miR-19b, hsa_miR_20a, hsa_miR-20b, hsa_miR-25, hsa_miR_92a, hsa_miR-93, hsa_miR-363, miR-363-5p, and miR-485-5p, TaqMan® Control Assay RNU48, TaqMan® Pri-miRNA Assay (hsa-pri-miR-106a, hsa-pri-miR-106b, hsa-pri-miR-17, hsa-pri-miR-25, hsa-pri-miR-92a-1, and hsa-pri-miR-92a-2) and TaqMan® Gene Expression Assays for TBP. Levels of expression miRNA were normalized to that of a small nucleolar RNA, C/D box 48 (RNU48). An internal control was also used to normalize between different plates. The qRT-PCR reaction was performed according to the manufacturer's protocol using the Stratagene MX 3005 qRT-PCR instrument (Agilent Technologies, Santa Clara, CA, USA). All results were analyzed using multiple experiment analysis applying a common threshold. All RNA samples were analyzed using both technical and biological triplicates.

### Statistical analysis of qRT-PCR data

The GenEx program (MultiD Analysis AB, Gothenburg, Sweden) was used to calculate fold-changes in levels of expression and to evaluate the significance of difference between Ct values. Levels of expression were normalized using the gene encoding the TATA-binding protein (*TBP*) as house-keeping. The non-parametric Mann–Whitney test was used to test differences between groups. Two-sided *P* ≤ 0.05 were considered statistically significant. Results are presented as means with SD indicated.

### Bioinformatic association of differentially expressed microRNAs with cellular functions

Bioinformatic analysis of miRNAs found to be differentially expressed was carried out using Ingenuity Pathways Analysis software (IPA) (Ingenuity Systems Inc., Redwood City, CA, USA, http://www.ingenuity.com). The principles of the algorithms involved were reviewed by Kramer et al. (2014).

## Results

### Effects of transfection with miR-18a, miR-19a-, miR-20b-, miR-92a-, miR-363-3p or miR-363-5p mimic on proliferation of cultured carcinoma cells

**Figure 2:**
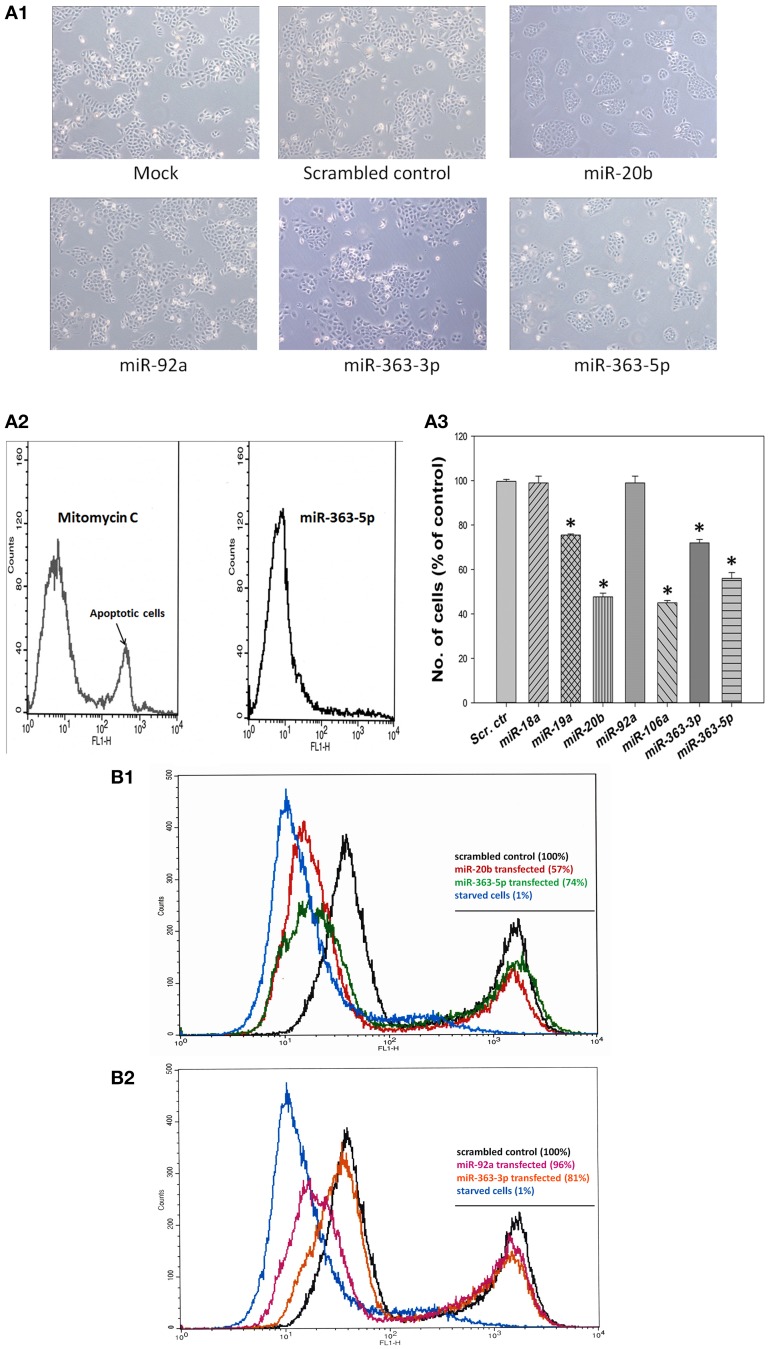
Effects of transfection with mimic for miR-19a, miR-20b, miR-92a, miR-363-3p (miR-363), or miR-363-5p (miR-363^*^) on cell densities of cultured human squamous carcinoma (E10) cells. (A) Cells from the carcinoma cell line E10 was transfected with different mirNA mimics (miR-19a, miR-20b, miR-92a, miR-363-3p, or miR-363-5p). Typical micrographs of cells subjected exposed to transfection medium only (Mock), or transfected with scrambled control RNA, or with one of the mirNA-mimics are shown in **(A1)**. Typical results obtained using the Single Channel Annexin V/Dead Cell Apoptosis Kit with cells transfected miR-363-5p-mimic showing no evidence of induced apoptosis. E10 cells incubated with mitomycin C to induce apoptosis served as positive controls for apoptosis **(A2)**. Cells counts following transfection with scrambled control or with mimic for miR-20b, miR-92a, miR-363-3p, or miR-363-5p (means derived from three separate transfections with SD indicated, **A3**). Levels of significance were computed as described in Materials and Methods using the Mann-Witney test. Means significantly different from the scrambled control mean are indicated by (^*^). **(B)** Panel presents typical results for cellular proliferation of E10 cells which 72 h earlier had been transfected with scrambled control, miR-20b - and miR-363-5p mimic **(B1)**, scrambled control, miR-92a mimic and miR-363-3p mimic **(B2)**. E10 cells incubated for 72 h in a serum free medium (starved cells) were used as positive control. Cellular proliferation was assayed using the Click-iT EdU Alexa Fluor® 488 kit. The numbers in parenthesis represent the area under the high intensity fluorescence peak expressed as % of that under corresponding peak for scrambled control. Serum starved E10 cells are included as positive control showing complete inhibition of DNA replication. Further experimental details are otherwise given in Materials and Methods.

Cultured human squamous oral carcinoma cells (E10) exhibited both altered morphology and diminished proliferation at 72 h after transfection with mimic for either miR-20b, or miR-363-3p, or miR-363-5p. These cells appeared less elongated than those of the other cultures investigated (Figure 2A1). In cultures transfected with mimics for miR-20b or miR-363-5p the number of cells were about 55 and 75%, respectively, of that found in scrambled control (Figure 2A3). Neither significantly decreased number of cells, nor altered cell morphology, were found with cells transfected with miR-18a - or miR-92a mimic (Figure 2A3). In cultures transfected with miR-363-3p mimic less apparent changes in cellular morphology were observed (Figure 2A1), even though the number of cells were about 70% of that of the scrambled control (Figure 2A3).

Assay of apoptosis revealed that no detectable stimulation of apoptosis had occurred following transfection with miR-363-5p mimic (Figure 2A2). Similar results were found with other mirNA-mimic causing decreased cell density (results not shown). Results presented in Figure 2 suggest that the significantly decreased cell densities observed with transfectants for mimic of miR-19a, miR-20b -, mir-106a, miR-363-3p -, and miR-363-5p were caused by diminished proliferation. Assay of proliferation using the Click-iT EdU Alexa Fluor® 488 assay showed a markedly decreased fraction of these cells exhibiting high intensity fluorescence (Figures 2B1,B2). The fraction of cells exhibiting high intensity fluorescence ranged from 57–81% of that found in the corresponding scrambled control peak. Serum starved cells were used a positive control demonstrating virtual absence of DNA replication in such cultures (Figures 2B1,B2). This demonstrates that DNA replication is decreased in these cells (Diermeier-Daucher et al., 2009). The lower value (57%) was found with cells transfected with miR-20b mimic, also exhibiting the larger decline in cell density (Figure 2A3). The 4% decrease in the high intensity fluorescence peak observed with transfectants for miR-92a mimic was presumably too small to cause a detectable change in cell density (Figure 2A3).

A markedly decreased number of cells was also observed with cultured keratinocytes and HaCaT cells transfected with miR-363-5p mimic (76 ± 2% and 74 ± 2% (*n* = 4) of scrambled control, respectively) suggesting that this phenomenon is not restricted to cultured E10 cells.

### Profiling of miRNAs which were differentially expressed in E10 cells transfected with miR-19a-, miR-20b-, miR-92a-, or miR-363-5p mimic

Microarrays were used to profile miRNAs in cultured human oral squamous carcinoma cells (E10) 72 h after transfection with mimics for miR-19a -, miR-20b -, miR-92a -, or miR-363-5p mimic or with scrambled control. Studies with miR-363-3p were not included as miR-363-3p has been shown to have an anti-proliferative effect (Sun et al., 2013).

**Figure 3:**
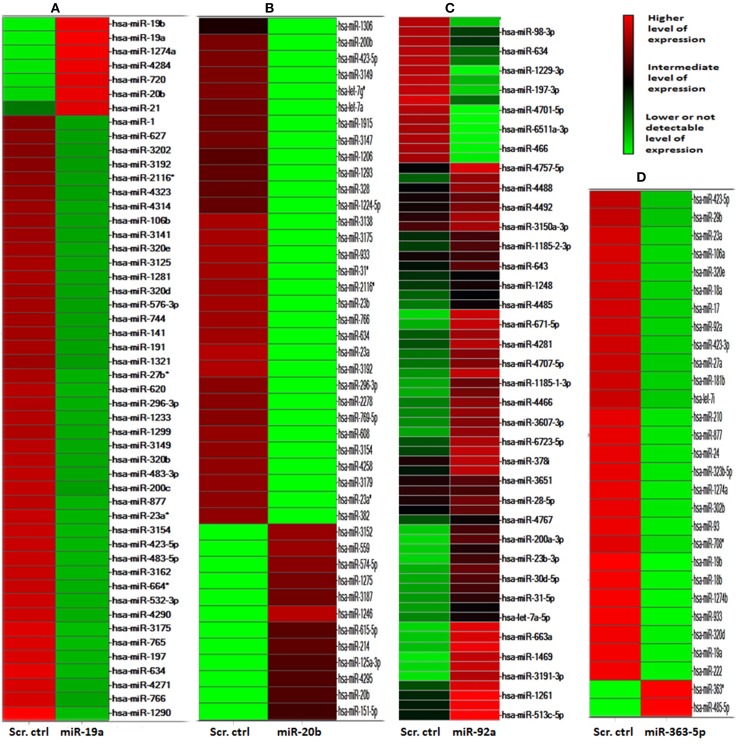
**MicroRNAs differentially expressed in cultured human squamous carcinoma (E10) cells transfected with scrambled control, miR-19a -, miR-20b -, miR-92a, or miR-363-5p- mimic**. The microRNA transcriptome of E10 cells transfected with miR-19a -, miR-20b -, mir92a -, or miR-363-5p mimic were individually compared against transcriptome from scrambled control transfectants. ANOVA (*P* ≤ 0.05) was used for the isolation of the 53 mirNA differentially expressed in transfectants with miR-19a mimic **(A)**, 43 miRNAs in miR-20b transfectants **(B)**, 61 miRNAs in miR-92a transfectants **(C)** and 29 miRNAs in transfectants with miR-363-5p mimic **(D)**. Each cluster was separately subjected to hierarchical clustering yielding the heat diagrams shown in the figure. The heat-map was computed using Spotfire DecisionSite for Microarray analysis as described in Materials and Methods. The color scale indicates fluorescence intensities. The results were obtained by microarray analysis of mirNA transcriptomes using four separate transfections for each mirNA mimic and four scrambled control transfections as described in Materials and Methods.

The heat-map presented in Figure 3 resulted from hierarchical clustering of 53, 43, 61 and 29 miRNAs found differentially expressed (*p* ≤ 0.05) in E10 cells transfected with mimic for miR-19a (A), miR-20b (B), miR-92a (C), or miR-363-5p - (D), respectively. With miR-19a mimic transfectants (A) 46 of the 53 miRNAs exhibited significantly decreased expression, miR-19a, miR-19b, and miR-20b being among the seven mirNA with significantly increased expression. Similarly, with transfectants for miR-363-5p mimic 27 of 29 miRNAs exhibited significantly decreased expression (about 50% of the level found in scrambled controls), only miR-363-5p and miR-485-5p exhibited increased (some 40-fold) expression (D). In contrast, with miR-20b mimic 31 of 43 differentially expressed miRNAs exhibited decreased expression (B). As expected, high levels of expression of miR-19a/miR-19b, or miR-20b, or miR-363-5p were found in cells transfected with mimic for miR-19a, or miR-20b - or miR-363-5p, respectively. Only in cells transfected with miR-19a mimic a high level of miR-20a was detected (A). One single mirNA, miR-423-5p, was found differentially expressed (decreased by about 50%) in transfectants for either miR-19a -, miR-20b -, or miR-363-5p mimic.

In cells transfected with miR-92a-mimic a distinctly separate population of miRNAs were found differentially expressed, not containing any mirNA encoded by any one of the three paralogous clusters (C).

### Changes in levels of expression of five selected genes and in levels of the corresponding proteins

Results obtained using qRT-PCR showed that ATF1, KRT14, KRT15, and PSMB6 were differentially expressed in miR-363-5p transfectants and AFT1 and PSMB6 in miR-20b transfectants (Table 1). Although EGFR was not differentially expressed (Table 1), it was found to be targeted by miR-20b and was therefore also used for Western blotting.

**Table 1:** **Relative changes in levels of five selected mRNAs following transfection of E10 cells with miR-20b - or miR-365-3p mimic**.

**Gene**	**Fold change in level of mRNA (transfected/scrambled control)**	
	**Transfected with miR-20b mimic**	**Transfected with miR-363-5p mimic**
ATF1	0.8 ± 0.1	0.5 ± 0.1
EGFR	0.9 ± 0.1	0.9 ± 0.03
KRT14	1.0 ± 0.1	0.2 ± 0.02
KRT15	1.1 ± 0.1	0.3 ± 0.02
PSMB6	0.5 ± 0.02	0.1 ± 0.01

Levels of mRNAs encoded by AFT1, EGFR, KRT14, KRT15, or PSMB6 were assayed using qRT-PCR with total RNA isolated from E10 cells transfected with scrambled control or miR-20b - or miR-363-5p mimic. The tabulated mean values (with SD indicated) were derived from at least three biological replicates. These five mRNAs were predicted to be targeted by miR-20b or miR-363-5p using MicroCosm, IPA or SVMicro software. Experimental details are otherwise given in Materials and Methods Section.

**Figure 4:**
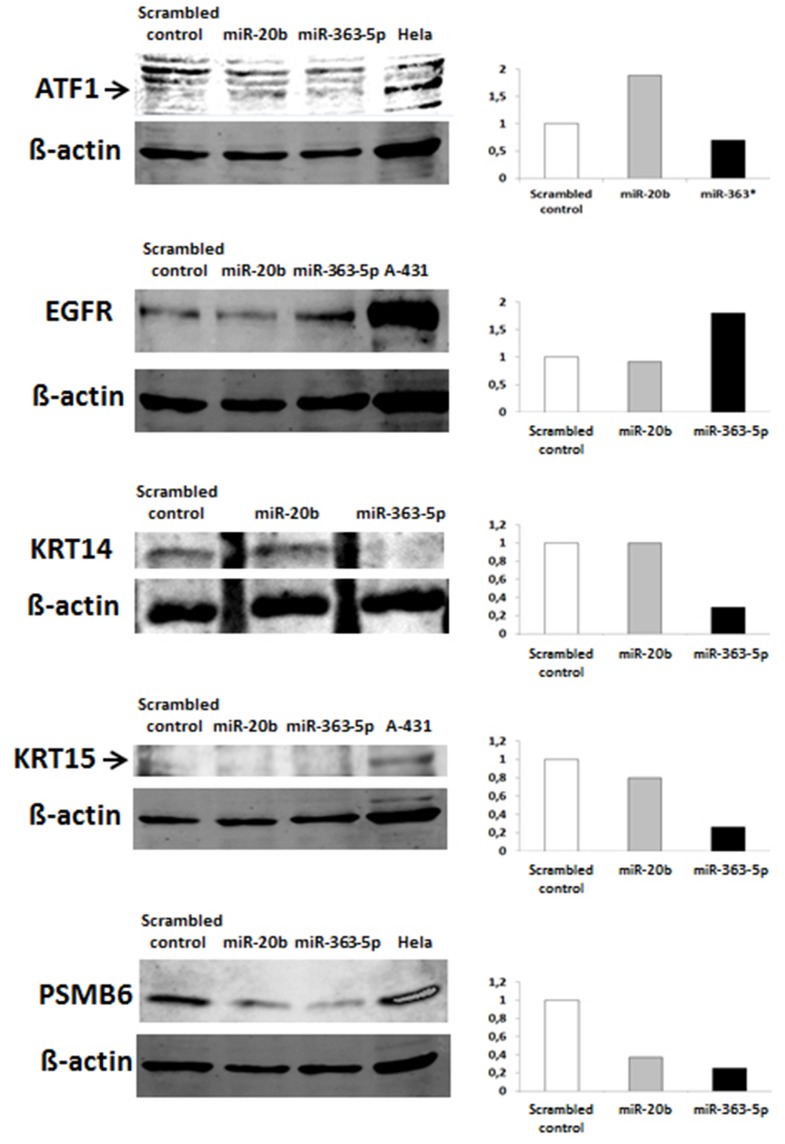
**Levels of selected proteins in cultured human squamous carcinoma cells (E10) transfected with miR-20b - or miR-363-5p mimic**. Effects of transfection of E10 cells with miR-20b - or miR-363-5p mimic on cellular levels of the proteins shown in the figure were investigated using Western blotting. Typical Western blot images are shown; the histograms showing integrated fluorescence intensities of the band of protein with molecular weight corresponding to that of the protein being investigated. The level of KRT15 protein in E10 cells is low, hence uncertain values for quantitation of this protein. Experimental details are otherwise presented in Materials and Methods.

The QRT-PCR results suggest that changes in levels of mRNAs (Table 1) are mostely in agreement with results from Western blotting (Figure 4). In cells transfected with miR-363-5p mimic, however, and increased level of EGFR protein was observed (Figure 4), suggesting that the level of EGFR protein is also regulated at the level of translation.

### Significant associations between differentially expressed microRNAs and cellular functions

**Figure 5:**
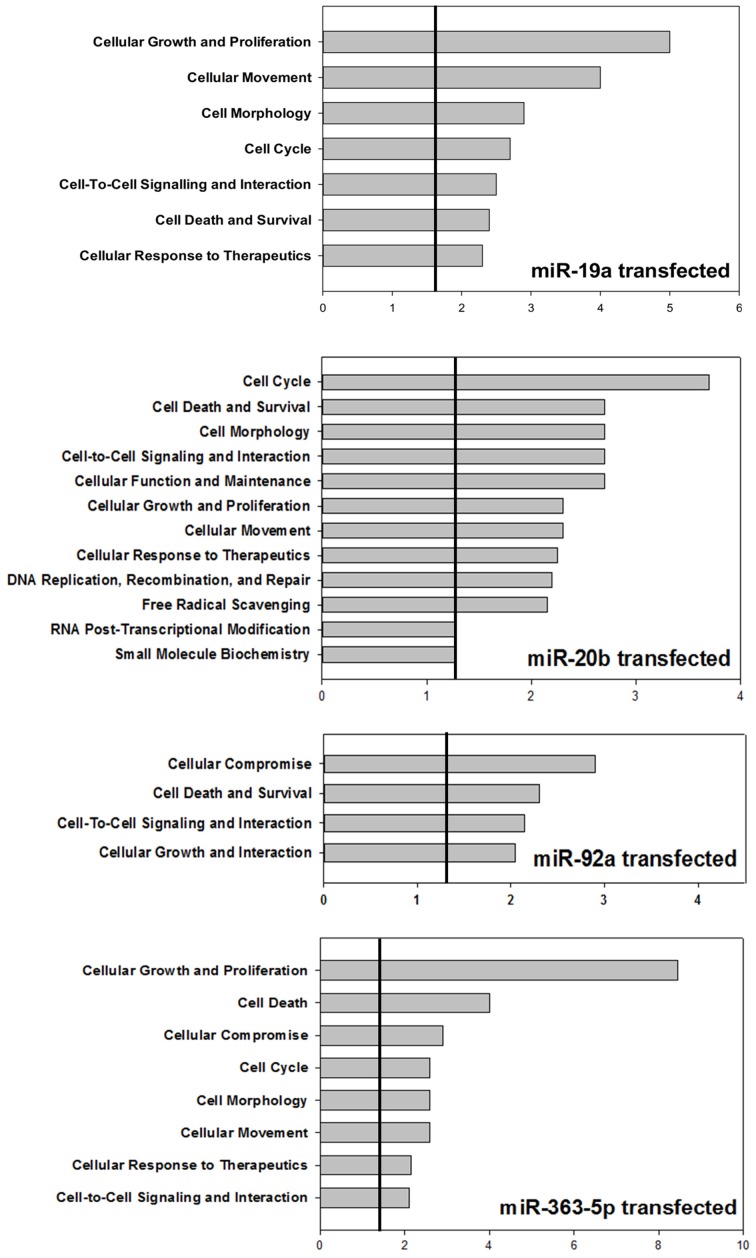
**Bioinformatic analysis of miRNAs found differentially expressed in cultured human squamous carcinoma cells (E10) transfected with miR-19a -, miR-20b -, miR-92a -, or miR-363-5p mimic**. The miRNAs (53, 43, 61 and 29) found differentially expressed following transfection with miR-19a - miR-20b -, miR-92a -, or miR-363-5p mimic were subjected to bioinformatic analysis using Ingenuity Pathways Analysis. The cellular functions significantly (*p* ≤ 0.05) associated with these mirNA are illustrated using histograms. The horizontal lines indicate the threshold at a level of significance when *p* = 0.05. The level of significance was computed as the Fisher's Exact Test *p*-value. Experimental details are otherwise described in Materials and Methods.

Ingenuity Pathways Analysis was used to establish associations between clusters of miRNAs and cellular functions. For cells transfected with mimic for miR-19a, miR-20b, or miR-363-5p (Figure 3) differentially expressed miRNAs yielded highly significant associations to, e.g., “Cell Cycle,” “Cell Death,” and “Cellular Growth and Proliferation” (Figure 5). In contrast, with miRNAs found differentially expressed following transfection with miR-92a mimic (Figure 3) these associations were not found (Figure 5). The miRNAs, and seed sequences, associated with the various cellular functions are presented in Table 2.

**Table 2:** **Differentially expressed miRNAs and significantly associated cellular functions**.

**Category**	***p*-Value**	**Molecules**
Cellular growth and proliferation	3,70E-09	miR-17-3p (and other miRNAs w/seed CUGCAGU), miR-17-5p (and other miRNAs w/seed AAAGUGC), miR-18a-5p (and other miRNAs w/seed AAGGUGC), miR-19b-3p (and other miRNAs w/seed GUGCAAA)
	8,15E-07	let-7a-5p (and other miRNAs w/seed GAGGUAG), miR-17-3p (and other miRNAs w/seed CUGCAGU), miR-17-5p (and other miRNAs w/seed AAAGUGC),miR-18a-5p (and other miRNAs w/seed AAGGUGC), miR-19b-3p (and other miRNAs w/seed GUGCAAA), miR-24-3p (and other miRNAs w/seed GGCUCAG), miR-29b-3p (and other miRNAs w/seed AGCACCA)
	3,82E-04	miR-221-3p (and other miRNAs w/seed GCUACAU), miR-23a-3p (and other miRNAs w/seed UCACAUU)
	2,46E-03	let-7a-5p (and other miRNAs w/seed GAGGUAG),miR-29b-3p (and other miRNAs w/seed AGCACCA)
	3,56E-03	let-7a-5p (and other miRNAs w/seed GAGGUAG),miR-17-5p (and other miRNAs w/seed AAAGUGC), miR-221-3p (and other miRNAs w/seed GCUACAU), miR-23a-3p (and other miRNAs w/seed UCACAUU)
	4,86E-03	miR-17-5p (and other miRNAs w/seed AAAGUGC)
	8,49E-03	miR-221-3p (and other miRNAs w/seed GCUACAU)
Cell death	1,01E-04	let-7a-5p (and other miRNAs w/seed GAGGUAG), miR-17-3p (and other miRNAs w/seed CUGCAGU), miR-17-5p (and other miRNAs w/seed AAAGUGC), miR-181a-5p (and other miRNAs w/seed ACAUUCA), miR-221-3p (and other miRNAs w/seed GCUACAU), miR-29b-3p (and other miRNAs w/seed AGCACCA)
	4,14E-04	miR-181a-5p (and other miRNAs w/seed ACAUUCA), miR-221-3p (and other miRNAs w/seed GCUACAU)
	2,43E-03	miR-17-5p (and other miRNAs w/seed AAAGUGC)
	3,65E-03	miR-181a-5p (and other miRNAs w/seed ACAUUCA)
	4,86E-03	miR-221-3p (and other miRNAs w/seed GCUACAU)
	5,86E-03	let-7a-5p (and other miRNAs w/seed GAGGUAG), miR-29b-3p (and other miRNAs w/seed AGCACCA)
	9,81E-03	miR-17-3p (and other miRNAs w/seed CUGCAGU), miR-181a-5p (and other miRNAs w/seed ACAUUCA)
Cellular compromise	1,22E-03	miR-17-5p (and other miRNAs w/seed AAAGUGC)
Cell cycle	2,43E-03	miR-17-5p (and other miRNAs w/seed AAAGUGC)
Cell morphology	2,43E-03	miR-17-5p (and other miRNAs w/seed AAAGUGC)
Cellular movement	2,43E-03	miR-17-5p (and other miRNAs w/seed AAAGUGC)
	9,70E-03	miR-221-3p (and other miRNAs w/seed GCUACAU)
	3,36E-02	let-7a-5p (and other miRNAs w/seed GAGGUAG)
Cellular response to therapeutics	4,86E-03	miR-17-5p (and other miRNAs w/seed AAAGUGC)
Cell-to-cell signaling and interaction	6,07E-03	miR-29b-3p (and other miRNAs w/seed AGCACCA)

Ingenuity Pathways Analysis was used to detect cellular functions significantly associated with miRNAs found differentially expressed following transfection with miR-19a - miR-20b -, miR-92a - or miR-363-5p mimic as shown in Figure 5. The Table presents the miRNAs found associated with the various cellular functions and their associated Fisher's Exact Test p-value. Experimental details are otherwise described in Materials and Methods.

### Effects of transfection of cultured squamous carcinoma cells with miR-19a -, miR-20b -, miR-92a -, or miR-363-5p mimic on levels of miRNAs encoded by the miR-17-92 cluster

**Figure 6:**
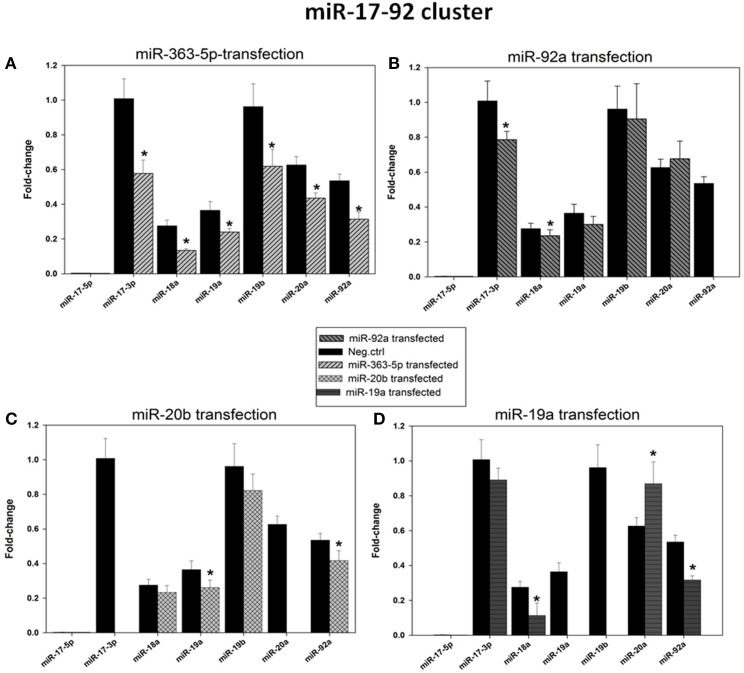
**Levels of expression of the microRNAs encoded by the miR-17-92 cluster in cultured human squamous carcinoma cells (E10) following transfection with miR-19a -, miR-20b -, miR-92a -, or miR-363-5p mimic**. Fractions of RNA enriched with respect to microRNA were isolated from cultured human squamous carcinoma cells (E10) transfected with mimic for miR-363-5p **(A)**, miR-92a - **(B)**, miR-20b - **(C)**, miR-19a - **(D)** mimic or scrambled control as shown in the Figure. Levels of expression of the various microRNAs encoded by the miR-17-92 cluster (shown in Figure) were assayed by qRT-PCR. Fold changes in expression and statistical parameters were calculated using the GenEx program. The plotted data represents mean values ± SD derived from at least three transfections. Median levels of expression significantly different from that of scrambled control are indicated by (^*^). Experimental details are otherwise given in Materials and Methods.

Microarray results suggested that levels of expression of five members of the miR-17-92 cluster (miR-17, miR-18a, miR-19a, miR-19b, and miR-92a) were significantly decreased only in cells transfected with miR-363-5p mimic (Figure 3). These findings were confirmed using qRT- PCR (Figure 6). The levels of expression of the mature miRNAs from this cluster were 50-70% of that observed in cells transfected with scrambled control (Figure 6).

Results presented in Figure 6 also show levels of expression of the various miRNAs of the miR-17-92 cluster after transfection with mimics for miR-19a, miR-20b or miR-92a. Although significantly decreased levels of expression of two or three mature miRNAs were observed, no systematic pattern is apparent. Results for miR-17 and miR-20a are uncertain as the assay is unlikely to selectively measure miR-17 and miR-20a due to their close homology to miR-20b (Figure 1), a problem compounded by the high level of miR-20b mimic in these transfected cells. Similarly, in cells transfected with miR-19a mimic selective assay of miR-19b is unlikely because these miRNAs differ by a single nucleotide only (Figure 1).

### Effects of transfection of cultured squamous carcinoma cells with miR-19a -, miR-20b -, miR-92a -, or miR-363-5p mimic on levels of miRNAs encoded by the miR-106a-363 or miR-106b-25 clusters

**Figure 7:**
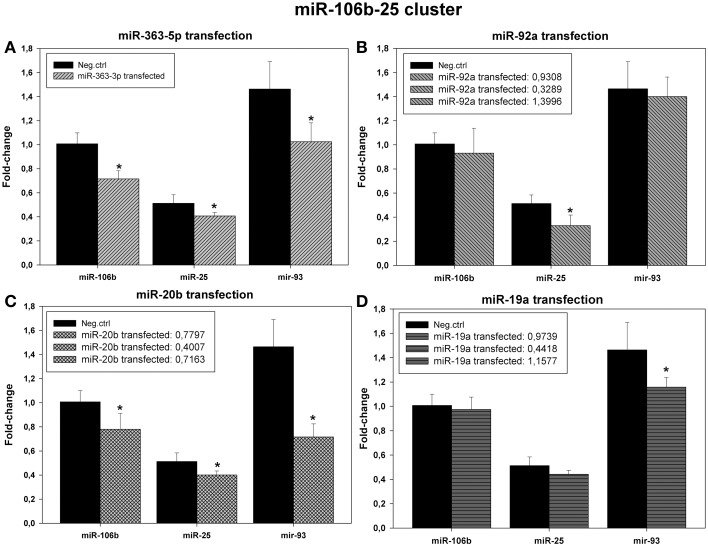
**Levels of expression of the microRNAs encoded by the miR-106b-25 cluster in cultured human squamous carcinoma cells (E10) following transfection with miR-19a miR-20b -, miR-92a -, or miR-363-5p mimic**. Fractions of RNA enriched with respect to microRNA were isolated from cultured human squamous carcinoma cells (E10) transfected with miR-363-5p **(A)**, miR-92a - **(B)**, miR-20b - **(C)**, miR-19a - **(D)** mimic or scrambled control shown in the Figure. Levels of the various microRNAs encoded by the miR-106b-25 cluster (shown in the Figure) were assayed by qRT-PCR. Statistical calculations were carried out using the GenEx program. Median levels of expression significantly different from that of scrambled control are indicated by (^*^). Experimental details are otherwise given in Materials and Methods.

Results from microarrays (Figure 3) and qRT-PCR (Figure 7) suggested that levels of expression of all members of the miR-106b-25 cluster (miR-106b, miR-25, and miR-93) were decreased in cells transfected with miR-20b mimic or with miR-363-5p mimic (Figures 7A,B).

In cells transfected with miR-19a - or miR-92a mimic no systematic changes in expression of miRNAs was apparent (Figures 7B,D).

**Figure 8:**
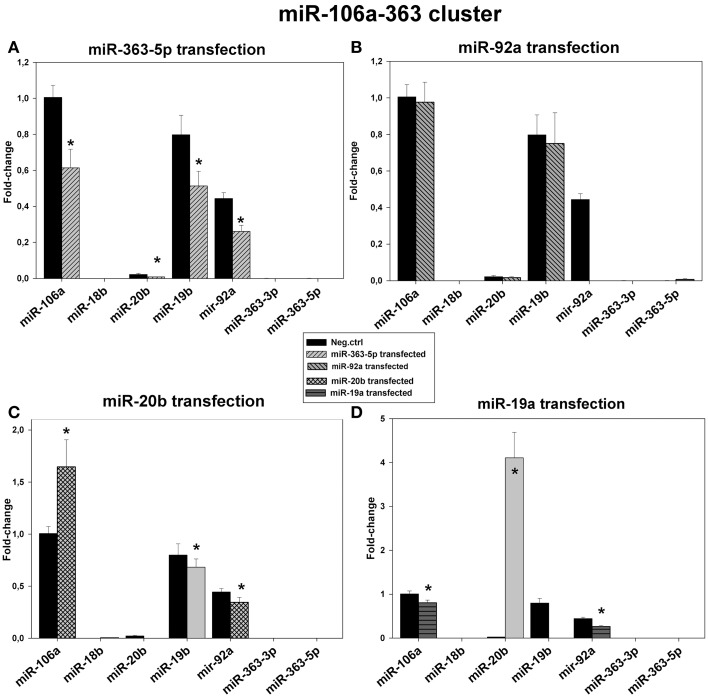
**Levels of expression of the microRNAs encoded by the miR-106a-363 cluster in cultured, human squamous carcinoma cells (E10) following transfection with miR-19a, miR-20b -, miR-92a -, or miR-363-5p mimic**. Fractions of RNA enriched with respect to microRNA were isolated from cultured human squamous carcinoma cells (E10) transfected with miR-363-5p **(A)**, miR-92a - **(B)**, miR-20b - **(C)**, miR-19a - **(D)** or scrambled control. Levels of the various microRNAs encoded by the miR-106a-363 cluster (shown in Figure) were assayed by qRT-PCR. Statistical calculations were carried out using the GenEx program. Median levels of expression significantly different from that of scrambled control are indicated by (^*^). Experimental details are otherwise given in legend in Materials and Methods.

Results presented in Figure 8 show relative levels of expression of the miRNAs encoded by the miR-106a-363 cluster after transfection of E10 cells with mimic for miR-19a-, miR-20b -, miR-92a -, or miR-363-5p. Only miR-19b, miR-20b, miR-92a, and miR-106a were detectable in these cells. A general decrease in all detected mirNA was found only in cells transfected with mirNA-363-5p mimic (Figure 8A). Transfection with miR-20b mimic led to 60% increase in the level of mirNA-106a (Figure 8C), while in cells transfected miR-19a mimic the level of miR-20b was increased 4-fold (Figure 8D) in agreement with microarray results (Figure 3). In all other transfected cells, including scrambled control, miR-20b was barely detectable. It is, however, possible that measured miR-19b and miR-92a were derived from the miR-17-92 transcript. Therefore, miR-20b and miR-106a may be the only mature microRNAs exclusively originating from the miR-106a-363 transcript.

Although the sequence of miR-20b differs from that of miR-106a by 3 bases (Figure 1) the specificity of assay of assay of miR-106a in cells transfected with miR-20b mimic is somewhat uncertain (Figure 8C). This point is more appropriate as regards assays of miR-19b in cells transfected with miR-19a mimic (Figure 8D), these two miRNAs differing by 2 bases (Figure 1).

### Effects of transfection cultured human squamous carcinoma cells with miR-19a -, miR-20b -, miR-92a -, or miR-363-5p mimic on expression of the primary transcripts pri-17-92, pri-106a-363, and pri-106b-25

**Figure 9:**
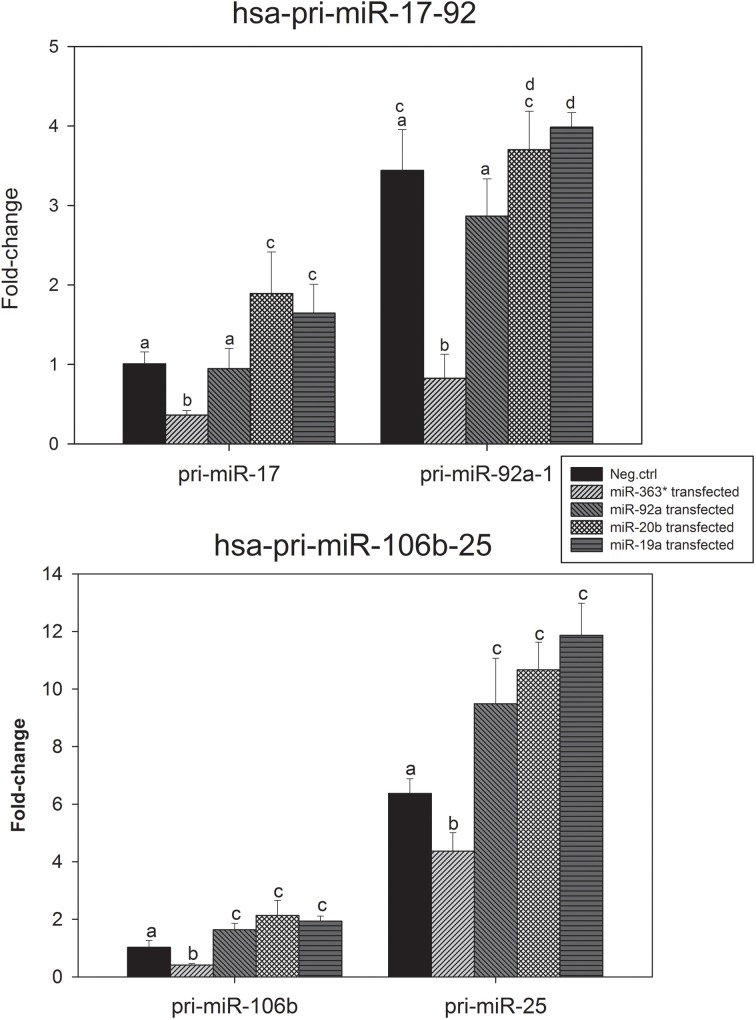
**Levels of the miR-17-92 - and the miR-106b-25 primary transcript in cultured human squamous carcinoma cells (E10) following transfection with mimic for miR-19a, miR-20b, miR-92a, or miR-363-5p**. Total RNA were isolated from cultured human squamous carcinoma cells (E10) transfected with miR-19a, miR-20b -, miR-92a - miR-363-5p mimic or scrambled control. The hsa-pri-miR-17, hsa-pri-miR-92a-1, hsa-pri-miR-106b and hsa-pri-miR-25 were assayed by qRT-PCR. The plotted mean fold changes in level of expression (with SD indicated) were derived from at least three separate transfections. Statistical calculations were carried out using the GenEx program. Mean values denoted by identical letters are not significantly different; those denoted by different letters are significantly different (*P* < 0.05). Experimental details are otherwise given in Materials and Methods.

The levels of expression of hsa-pri-miR-17, hsa-pri-miR-92a-1, hsa-pri-miR-106b, hsa-pri-miR-25, hsa-pri-miR-106a, and hsa-pri-miR-92a-2 were measured in E10 cells after transfection with miR-19a, miR-20b -, miR-92a -, or miR-363-5p mimic. The results for pri-miR-17-92 and pri-miR-106b-25 are presented in Figure 9.

Results in Figure 9A show the level of expression of hsa-pri-miR-92a-1 was higher than that of hsa-pri-miR-17 in all cells investigated. In cells transfected with miR-363-5p mimic levels of expression of both pri-miR-17 and pri-miR-92a-1 were significantly decreased. This correlates with the observed decrease in levels of mature miRNAs encoded by the cluster following transfection with miR-363-5p mimic (Figure 6A).

Transfection with miR-92a mimic had no significant effect on levels of expression of either of the pri-transcripts (Figure 9). Transfection with miR-20b mimic, however, significantly increased expression of pri-miR-17, while that of the pri-miR-92a-1 transcript was unaffected (Figure 9). Transfection with miR-19a mimic resulted in significantly increased expression of both pri-miR-17 - and pri-miR-92a-1 transcript (Figure 9).

Results presented in Figure 9 show that the level of expression of the pri-miR-25 transcript was higher than that of the pri-miR-106b transcript. In cells transfected with miR-363-5p mimic the level of expression of both of these transcripts were decreased. This correlates with the observed decreased expression of the mature miRNAs encoded by this cluster following transfection with miR-363-5p mimic (Figure 7A). In cells transfected with miR-19a -, miR-20b -, or miR-92a mimic, the primary transcripts exhibited significantly increased levels of expression (Figure 9).

The relative levels of primary transcripts from the three clusters were rather different, also in a 5′-/3′-specific manner. Using the level of expression of the pri-miR-17 (5′-end) transcript as reference, that of pri-miR-92a-1(3′-end) was 4.6 ± 0.7 fold higher and those of pri-miR-106b (5′-end) and pri-miR-25 (3′-end) were 8.4 ± 2.0 - and 52.1 ± 4.1-fold higher, respectively. Similarly, the level of expression of pri-miR-106a (3′-end) was 0.0003 ± 0.00006, while that of pri-miR-92a-2 (3′-end) was not detectable in any of the samples assayed (also in HTC116 and HT29 cells, results not shown). This likely explains why only miR-106a is detected in E10 cells.

## Discussion

An anti-proliferative effect has previously been observed for miR-19a (Qin et al., 2010), miR-106a (Yang et al., 2011) and miR-363-3p (Sun et al., 2013). Searching the literature revealed no such findings as regards miR-20b and miR-363-5p. Because overexpression of miR-17-92 - or miR-106a-363 cluster is associated with stimulated proliferation/cancer (Landais et al., 2007; Lu et al., 2007) the observed anti-proliferative effect found with miR-20b and 363-5p transfectants was unexpected. It may, however, be associated with the relatively low levels of miR-20b and miR-363-5p found in E10 cells (Figure 8).

The pleiotropic functions of the miR-17-92 cluster and its paralogs are perhaps their more striking feature (reviewed by Olive et al., 2010). Although high levels of expression are associated with cancer, information on specific effects of individual mirNA is invariably incomplete. The present findings provide new data as regards possible functions of individual miRNAs, while also suggesting that the mechanism regulating their expression is composite in nature. This is exemplified by the selective 50-fold increase in the level of the anti-proliferative miR-20b in transfectants for miR-19a-mimic (Figure 8D), which also exhibited about 25% decrease in cell density (Figure 2A3). Likewise, the more potent anti-proliferative miR-20b decreased expression miR-19a and miR-93 (Figures 6C, 7C) as well as increasing the level of miR-106a by about 60% (Figure 8C); changes consistent with an anti-proliferative effect (Qin et al., 2010; Fang et al., 2011). Results obtained with transfectants for miR-363-5p further illustrate the heterogeneity of effects on sibling miRNAs: only in these transfectants were levels of the sibling miRNAs from all three clusters found significantly decreased (Figures 6–9). Indeed, it is conceivable decreased proliferation here is associated with a generalized decreased expression of cluster miRNAs—in line with the association of high levels of expression of miR-17-92 cluster in proliferating tissues. The decreased levels of miRNAs can be associated with diminished transcription of the cluster genes as suggested by a general decrease in levels of both mature miRNAs and of corresponding primary transcript in transfectants for miR-363-5p mimic (Figures 6–9). The reason for the increased level of primary transcript found with other transfectants is not apparent.

In this study most the observed changes in levels of most miRNAs ranged from −2- fold to +5-fold, with a few exceptions. Changes in expression of miRNAs on this scale have been considered to be biologically significant (Hidaka et al., 2012; Van Iterson et al., 2013; Yamada et al., 2013). Western blotting results, with proteins selected from among mirNA targets, can be regarded to verify this as all exhibited altered levels in cells transfected with mimic for miR-20b or miR-363-5p (Figure 4).

Among miRNAs encoded by the miR-106a-363 cluster only miR-106a is expressed at detectable levels in E10 cells. All tested, non-expressed; miRNAs caused 30–50% inhibition of proliferation. It is therefore possible also non-expressed members of this clusters may exert an anti-proliferative effect in some populations of cells, hence their non-expression in carcinoma (E-10) cells.

Whether the distinct populations of differentially expressed miRNAs found in transfectants for miR-19a -, miR-20b -, or miR-363-5p mimic (Figure 3) reflect different ant-proliferative mechanisms remains to be established. It is, however, noteworthy, that miR-92a-transfectants (exhibiting no change in proliferation) yielded an entirely different population of miRNAs (these transfectants also rarely exhibiting significant changes in expression of any cluster miRNAs, Figures 6–9).

Only miR-423-5p was shared between the 30–50 differentially expressed miRNAs in cells transfected with mimic for miR-19a, miR-20b, or miR-363-5p. However, high levels of expression of miR-423-5p has been found in breast—(Van Der Auwera et al., 2010) and hepatocellular tumors (Murakami et al., 2013). Therefore, the decreased expression (about 50%) of miR-423-5p observed in these transfected cells is therefore in line with published findings and may have contributed to inhibition of proliferation. A similar case can be made as regards the 40-fold increase in the level of miR-485-5p in cells transfected with miR-363-5p mimic, as miR-485-5p has been shown to functions as a tumor suppressor in breast carcinoma cells (Anaya-Ruiz et al., 2013).

In spite of the diverse composition of the clusters of differentially expressed miRNAs in cells exhibiting inhibition of proliferation (Figure 3), the three clusters nevertheless yielded similar significantly associated cellar functions, e.g., “Cell Cycle,” “Cell Death,” and “Cell-to-Cell Signaling and Interaction.” This may be taken to indicate that different anti-proliferative mechanisms operate in the various transfectants, as also suggested by results from Western blotting analysis. The cluster derived from miR-92-transfectants, however, yielded no such significantly associations (Figure 5) lend support to the suggestion that the anti-proliferative mechanism entails altered expression of miRNAs. The precise molecular mechanisms involved, however, remains to be established.

### Conflict of interest statement

The authors declare that the research was conducted in the absence of any commercial or financial relationships that could be construed as a potential conflict of interest.

## Supplementary material

Supplementary Figure 1 **The Figure show micrographs of E10 cells transfected with scrambled control, miR-20b-, miR-363-3p or miR-363-5p mimic**. Both miR-20b and miR-363-5p transfectants showed altered morphology; cells appeared larger and less elongate than scrambled control cells. With miR-363-3p transfectants changes in morphology were less clear, the cells appearing more like those of the scrambled control.
