# Supplementary figures and images for: An investigation into anti-proliferative effects of microRNAs encoded by the miR-106a-363 cluster on human carcinoma cells and keratinocytes using microarray profiling of miRNA transcriptomes
Source: Front Genet. 2014 Aug 25;5:246. doi: 10.3389/fgene.2014.00246 (PMC4142865; doi:10.3389/fgene.2014.00246)

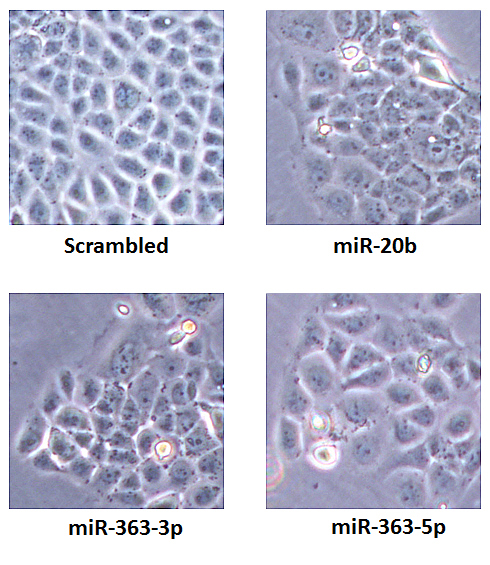

Supplement: Supplementary Figure 1 — The Figure show micrographs of E10 cells transfected with scrambled control, miR-20b-, miR-363-3p or miR-363-5p mimic. Both miR-20b and miR-363-5p transfectants showed altered morphology; cells appeared larger and less elongate than scrambled control cells. With miR-363-3p transfectants changes in morphology were less clear, the cells appearing more like those of the scrambled control. [file Image1.JPEG]
